# "Rehabilitation schools for scoliosis" thematic series: describing the methods and results

**DOI:** 10.1186/1748-7161-5-27

**Published:** 2010-12-24

**Authors:** Manuel D Rigo, Theodoros B Grivas

**Affiliations:** 1 Institute E. Salvá, Vía Augusta 185, 08021, Barcelona, Spain; 2 Department of Trauma and Orthopaedics, "Tzanio" General Hospital - NHS, Tzani & Afendouli str, 18536, Piraeus, Greece

## Abstract

The Scoliosis Rehabilitation model begins with the correct diagnosis and evaluation of the patient, to make treatment decisions oriented to the patient. The treatment is based on observation, education, scoliosis specific exercises, and bracing. The state of research in the field of conservative treatment is insufficient. There is some evidence supporting scoliosis specific exercises as a part of the rehabilitation treatment, however, the evidence is poor and the different methods are not known by most of the scientific community. The only way to improve the knowledge and understanding of the different physiotherapy methodologies (specific exercises), integrated into the whole rehabilitation program, is to establish a single and comprehensive source of information about it. This is what the *SCOLIOSIS J*ournal is going to do through the "Rehabilitation Schools for Scoliosis" Thematic Series, where technical papers coming from the different schools will be published.

## Background

Idiopathic Scoliosis can be defined as a complex three-dimensional deformity of the spine and trunk, which appears in apparently healthy children, and can progress in relation to multiple factors during any rapid period of growth. Specific prevention of idiopathic scoliosis is not possible, because its ultimate cause is unknown; however, there is a high consensus about the necessity to prevent curve progression. For most scoliosis specialists, conservative management of idiopathic scoliosis is related to bracing, and its main objective is to prevent curve progression to a more or less arbitrary critical point, where surgical correction would be indicated. Scoliosis specific rehabilitation is more comprehensive than this, and has gained an increased interest during the past several years.

Generally speaking, the objectives of scoliosis rehabilitation are to maintain function, and prevent symptoms in the short and long-term. More specifically, scoliosis rehabilitation's goal is to prevent curve progression, while trying to correct the spinal and trunk deformity in the long term, and to prevent the health related quality of life from deteriorating, while trying to improve it. Scoliosis Rehabilitation follows a model including:

1) Correct diagnosis and evaluation of the patient to make a treatment decision oriented to the patient.

2) Treatment is based on: observation (with a rational use of X-rays), education, specific physical exercises, and bracing.

3) Quality control and evaluation of the results.

Thus, scoliosis specific exercises are included in this model for many rehabilitation schools in several European countries, such as France, Italy, Germany, Spain, Poland, and others. However, the role of scoliosis specific physical exercises has been underestimated, due to a lack of evidence published in the English literature. In addition, because of an understandable lack of interest from a vast part of the medical community, and little support for research in this area, the specific methods applied for these rehabilitation schools are nowadays unknown. Consequently, most of the specialists in the world consider physical methods to be 'alternative medicine'.

No matter what name is given to any of those methods, and whether or not they have a more or less serious scientific, and/or empirical basis, they all tend to be considered in the same way. There is no distinction made between scoliosis specific exercises, general physical exercises, osteopathy, chiropractic, yoga, etc. Notwithstanding, in a recent comprehensive systematic review of the literature, Negrini S and collaborators have shown that, for a combination of some scoliosis specific physical exercises methods, the level of evidence has changed from 2a to 1b (Oxford EBM Centre) during the last five years [1]. Most of the results have been published by experienced teams using a multidisciplinary approach, where specific physical exercises methods with appropriate basic principles are used exclusively, or in combination with bracing, or surgery (pre- and post-).

The 2005 SOSORT consensus paper on physical exercises in the treatment of idiopathic scoliosis, at risk of brace treatment [2], reported that specialists in scoliosis physiotherapy agree on several features that can be regarded, currently, as standard features in the rehabilitation of scoliosis patients. These features include auto-correction in 3D; training in activities of daily living; stabilizing the corrected posture, and patient education. Most recognized rehabilitation schools involved in SOSORT use exercise programs based on these principles. Another important fact, consistent among the different scoliosis rehabilitation schools, is a multidisciplinary approach. The majority of proposed conservative treatment methods recognize that no isolated professional can be successful on a regular basis. Therefore, most recognized rehabilitation schools use a basic multidisciplinary working team, formed by one medical doctor (MD) with specialized knowledge, one physiotherapist (PT), and one orthotist (CPO). A psychologist may also be included, or necessary at times, although an experienced basic team can properly manage most of the patients. In other words, in scoliosis rehabilitation, like in many other rehabilitation issues, methods are not efficient by themselves, and in any case, failures may always happen.

According to the above mentioned 2005 SOSORT consensus paper, any methodology to treat IS with exercises should take in consideration the three-dimensional nature of IS. We would add to this the importance of the pathomechanism of progression, as prevention of curve progression is one of the main goals of the specific exercise. In this paper, we provide a short introduction to the three-dimensional nature of IS, and its pathomecanism of progression, both essential points in defining scoliosis specific exercises versus simply general physical exercise. We then invite the different rehabilitation schools to present, in a rational way, their methodologies and results.

## The three-dimensional nature of Idiopathic Scoliosis

Jean Dubousset wrote in 1992 [3]: *"The tri-dimensional nature of scoliosis was identified in the 19^th ^century. John Shaw recognized it in 1824, and clearly demonstrated that the apex of the deformity is lordotic, with the anterior column longer than the posterior column. In 1865, Adams also described the presence of lordosis in the thoracic apical region. With the arrival of radiology in 1895, the anatomical observations made by Shaw and Adams were quickly forgotten. The projections of the skeleton produced by X-rays were so attractive for doctors, surgeons, etc. that their thoughts were concentrated only on what was projected, the anteroposterior view, and rarely the sagittal view. As a result of this unidimensional approach, errors occurred in the use of instrumentation systems of the spine, creating the lumbar flat back syndrome, for example. This occurred throughout the world, in spite of the efforts made by Roaf and Somerville in their persistent description of the 3D nature of the scoliotic deformity. Dickson has recently underlined these findings of his British colleagues. In France, Rene Perdriolle was a pioneer in promoting the reality of the tri-dimensional nature of the deformity"*. During the second half of the twentieth century, a series of orthoses were developed within this one-dimensional, or two dimensional maximum, context by people who at least considered rotation. These braces are still used as standard scoliosis treatment in some countries, even though many of these brace concepts were later related to the thoracic and lumbar flat back syndrome [4-9].

Scoliotic deformity can be described as a *'series of vertebral segments placed in extension, or lordosis, which deflect and axially rotate towards the same side' *(Dubousset 1992). According to Dubousset, rather than a succession of lateral deviations, idiopathic scoliosis represents the combination of torsional regions joined by junctional zones. The Scoliosis Research Society (SRS) recognizes two meanings to this term torsion [10]: The first is mechanical torsion, which refers to the torsional deformity of the column considered as a plastic structure. Mechanical torsion affects the disc (intervertebral torsion) as well as the vertebra (intravertebral torsion) [11]. The second meaning is geometrical torsion. Geometrical torsion is defined as the 'tortuosity' of the spine considered as a line in space. The column changes its physiological shape in the frontal, transverse and sagittal planes, adopting extremely diverse anatomoradiological patterns. Torsional forces produce both mechanical and geometrical torsion. Geometrical torsion is related to translation of the apical vertebra. Several authors have described the evolution of a right thoracic scoliosis as a torsional phenomenon that translates the apical vertebra first ventro-lateral and further latero-dorsal, away from the upper end vertebra (UEV) [12,13]. Consequently, the scoliotic spine initially becomes more or less lordotic, from any given configuration, and further develops as a paradoxical kyphoscoliosis. It must be differentiated between geometrical lordosis and structural lordosis. Morphologically, IS is a fixed lordotic deformity of the spine; however the degree of this anatomical lordosis is variable. On the other hand, the scoliotic spine can adopt highly variable sagittal profiles from a geometrical lordosis to a paradoxical kyphosis. Obviously, nowadays few scoliosis cases progress to reach this last condition unless the lordotic structural component is minimal, and the original sagittal geometry was already normo or hyper-kyphotic. On the other hand, it must be pointed out that several authors have concluded that the lordotic component is an essential component for the pathogenesis of idiopathic scoliosis as a compensatory mechanism, but is not an aetiological factor. The main paper claiming this was conducted by Grivas TB et al [14]. In this study the lateral spine profile of mild (10°-20°) scoliotic curves was found to be similar to the lateral spine profile of their healthy controls. This study, as others [15], provides evidence that thoracic hypokyphosis, by facilitating axial rotation, could be viewed as being permissive (a compensatory mechanism), rather than as aetiological factor, in IS pathogenesis.

Following with Dubousset's description, scoliosis correction may be achieved through detorsional forces, with the intention of better aligning the column in the frontal plane, and normalizing the sagittal configuration of the spine. Postural three-dimensional correction, during scoliosis specific exercises, means that the patient will try, with and without external assistance, to achieve the best possible frontal, transversal and physiological sagittal alignment, before producing any active muscle activation to stabilize the correction. The three-dimensional correction must be performed in combination, and synchronized in all three planes; not during a set of sequential exercises which approach the correction by isolating plane by plane (for example: one exercise to correct in the frontal plane; a second exercise to correct in the transversal plane, and a third exercise to correct in the sagittal plane).

## The pathomechanism of progression

R.G. Burwell, in Pediatric Rehabilitation, published a complete revision on this topic, later revisited in the ICL book of the SOSORT [16,17], and more recently in a full paper published in SCOLIOSIS [18]. Burwell, in agreement with the biphasic concept, concluded that *"there is a view that there are two types of pathogenesis factors for idiopathic scoliosis: initiating (or inducing) factors and those that cause curve progression*". He deeply explores the description of these factors: *"progressive AIS, mainly affecting girls, is generally attributed to relative anterior spinal overgrowth from a mechanical mechanism (torsion) during the adolescent growth spurt." *There are some biological, morphological, neuromuscular and biomechanical susceptibilities, but four main factors have been well established as progression factors: Asymmetrical loading of the spine, vertebral growth modulation, spine slenderness and growth potential. In the above mentioned susceptibilities, the intervertebral disc could be included as an additional morphological factor involved in the progression of an IS curve. Its role will also be examined later as a fifth factor.

The four factors are related to the "vicious cycle concept" described by Stokes [19] or "the growth-induced torsion concept" modified by Burwell from Stokes. Stokes showed that an imposed vertebral deformity could be corrected by reversing the load used to create it. *'This implies that the principles of the Hueter Volkmann law are applicable to the correction of an existing vertebra deformity providing there is sufficient residual growth'*. They also showed that '*when the external loading is removed, growth rates return to normal, demonstrating that growth was not permanently affected by previously applied external loading'*. The results of their study have implications in the design, use, and effectiveness of bracing as well as physical therapy methods in the treatment of IS. They suggest that '*if sufficient force is applied to the vertebra the progression of a scoliosis could be arrested, or even reversed'*. Whether or not the progression of an established scoliotic deformity is secondary to asymmetric loading, correction of the deformity using the principles of Hueter-Volkmann law is possible as long as there is sufficient residual growth. Other studies [20,21] have demonstrated the feasibility of the modeling approach achieving at the same time a complete representation of the scoliotic spine.

Inciting the first factor, in human scoliosis, asymmetrical loading could result from:

- *The effect of gravity*

- *Muscle action*

- *Lordosing reactive forces*

- *Human gait*

- *Growth induced torsion*

### The effect of gravity

Gravity promotes progression in any curvature exceeding a critical point. Axial forces produced by gravity become asymmetric in a scoliotic spine. In the presence of a structural lordo-scoliosis, asymmetric loading produces a lateral force vector which increases translation with coupled vertebral rotation. The 'vicious cycle' model explains how lateral deviation increases vertebral and disc deformity. Haderspeck and Shultz [22] (1981) studied the muscle, and body weight, actions in scoliosis progression, and their conclusions were reviewed by Shultz in a paper [23] published in the proceedings of the International Symposium on 3D Scoliotic Deformities joined with the VIIth International Symposium on Spinal Deformity and Surface Topography (Montreal 1992). Schultz summarized that *'application of superior body segment weights were capable of causing substantial increases in Cobb measures. Body weight application effects were influenced by initial spine morphology. The Cobb measure changes produced were to some extent dependent on whether, and how, the trunk was restored to its upright position after the given force application'*. Thus, it seems that the consequences of the need for the trunk structures to support the weight of the body segments superior to them would be obviously different, when comparing passive scoliotic posture and active 3D corrected posture, at least theoretically.

### Muscle action

Although it has been studied at large, there is still controversy as to whether, or not, a muscle disease is a primary factor in the etiopathogenesis of IS. However, it seems clear that IS produces a secondary muscle imbalance, one of the most important factors in the progression of the deformity [24]. Recent studies have shown, in the natural history of IS, that spinal growth velocity and electromyography ratio at the lower end vertebra are prominent risk factors of curve progression [25,26]. The asymmetric muscle activity has been clearly associated with increased axial rotation, lateral deviation and decreased kyphosis.

### Lordosing reactive forces

This is related to the bi-planar theory of Dickson et al. [27]. In the presence of a lateral deviation, and/or axial rotation, combined with an asymmetrical shortening of the dorsal elastic structures, any flexion effort is converted into a lordotic force. Due to reflex mechanisms, flexion movements of the spine provoke tension on the dorsal elastic structures, which produce a reactive asymmetrical concentric force, increasing lordosis, and secondarily, axial rotation and lateral deviation as well.

### Human gait and torsion

According to the Nottingham thoracospinal concept, torsional forces are produced during gait [28]. When examining gait dynamics, axial pelvis-lower spinal rotation is counteracted by axial upper spinal counter-rotation. Burwell called it the 'dinner plate tent-pole' concept where the pelvis is likened to a dinner plate, and the spine to a flagpole or tent-pole. The gap between the upper spine and the lower spine represents the transitional point, above which axial rotation is in the direction opposite to that below. In the thoracic spine, rotation is maximal about T7, and minimal at the lower three levels.

### Growth induced torsion

Progression in AIS has been attributed to a relative anterior spinal overgrowth - *RASO *- [29] which causes a growing induced torsion. Although the existence of *RASO *is widely accepted, the question of which mechanism(s) is related is still controversial. No matter what causes RASO, according to Burwell's model, lordoscoliosis formed by growing torsion is what causes eccentric loading, eccentric growing, and vertebral and disc deformity. This is as a more complex interpretation of the 'vicious cycle' model, and is closely related with the three-dimensional nature of the deformity.

### The role of the intervertebral disc in the progression, and regression, of a scoliotic curve

It has been reported that in mild scoliotic curves, when the deformity is initiating, the intervertebral disc (IVD) is found wedged, but the vertebral body is not. The spine is deformed first at the level of the IVD, due to the increased plasticity of the IVD, in the way of either torsion, or wedging, as an expression of other initiating factors that may result in idiopathic scoliosis (IS) [30,31]. This was also verified three years later by Will et al 2009 [32]. The IVD contains the aggrecans of glycosaminoglycans (GAGs), which imbibe water through the so called Gibbs-Donnan mechanism. The highest concentration of aggrecans is in the nucleus pulposus (NP), where they are entrapped in a type II collagen network [33]. There is an increased collagen content in the NP of AIS IVD, which is maximal at the apex of the curvature. Furthermore, in the scoliotic spine the NP in the IVD is displaced towards the convex side of the wedged interspaces [34]. Differences also exist in the collagen distribution, between the concave and convex sides of the scoliotic annulus fibrosus in AIS, with depleted levels in the former compared to the latter [35]. Composing all the above findings, it has been suggested that the imbibed water, mainly in the apical IVD, but also in the adjacent discs above and below it, must be in a greater amount in the convex side than in the concave [30,31]. This asymmetrical pattern of the water distribution in the scoliotic IVD, in association with the diurnal variation in the water content of IVD - "swelling" (during night time) - and "shrinkage" (for the period of day time under the application of the body load during the upright posture), [36] imposes asymmetrical, convex-wise, concentrated cyclical loads to the IVD, and the adjacent immature vertebrae (vertebral growth plates) of the child during the 24 hour period. The convex side of the wedged IVD sustains greater amount of expansion than the concave side, leading to the sequelae of asymmetrical growth of adjacent vertebrae (Hueter- Volkmann law), due to asymmetric diurnal variations in loading during the 24 hour period.

The strong correlation between lumbar Lower InterVertebral Disc Wedging (LIVDW) and thoracic Cobb Angle (CA) [30,31] implicates the important role of the lumbar spine, and particularly that of the lumbar LIVDW, to the progression of the scoliotic curve, as the lumbar IVDs are significantly larger. The correlations found [30,31] imply that the apical intervertebral disc wedging, through the proposed mechanism, seems to be an important contributory factor in the progression of IS curves, emphasizing the role of the apical intervertebral disc in IS pathogenesis. Consequently, the response of bone growth to asymmetrical loading follows an asymmetrical pattern, and gradually can enhance the correction of the deformity, if optimal corrective forces are applied with a specific physiotherapy program [37].

## General principles for conservative treatment

According to the above described factors, we suggest that conservative treatment should be based on several general principles:

1. Prevention of asymmetric compressive forces related to passive posture

2. Reduction of the secondary muscle imbalance

3. Prevention of the lordosing reactive forces (passive posture, repeated forward bending movements)

4. Prevention of asymmetric torsional forces from gait

5. Production of dynamic detorsional forces involving breathing mechanics.

All of these principles are directed to reverse the 'vicious cycle' into a 'virtuous cycle'. However, this could not be achieved without considering the constant interaction between the growing skeleton and the neural system. Burwell and collaborators [17] have defined this interaction, by taking an idea expressed originally by Nachemson, the '*neuro-osseous timing of maturation -NOTOM - system escalators'*. Burwell, and collaborators, describe a collective model for adolescent idiopathic scoliosis pathogenesis, which involves abnormality of the escalators of a normal neuro-osseous timing of maturation system as a central concept: *'In normal growth and maturation, the two polarized processes are synchronous, and symmetric, linked through sensory input and motor output. In AIS pathogenesis, the polarized processes are asynchronous, with asymmetry on one, or both, of the processes'*. Abnormality of these two polarized processes is associated with the initiation, and progression, of right thoracic AIS, with relative spinal overgrowth and torsion. In this model, the central neural system's (CNS) postural maturation delay, and/or asymmetry, fails to control initiating scoliosis. The asymmetric input will change normal 'reference corporal schema' at the CNS, becoming a 'scoliotic corporal schema' with its abnormal asymmetric motor output. Whether, or not, this abnormal asymmetric motor output comes from a primary, or secondary, neurological failure, the fact is that it promotes, and increases, the asymmetric muscle function, which can be considered a progression pathomecanism factor. This represents a parallel 'neural vicious cycle' related to the neurological function, which would empower the first described 'growth-skeletal vicious cycle'.

## General Principles of Specific Physical Therapy Methods

Specific physical therapy methods should consider two main premises in the treatment of IS; the 3D nature of the condition, and the skeletal, and neurological, pathomechanism of progression. The main objective of physical therapy should be to convert the 'vicious cycle' into a new 'virtuous cycle', where deforming forces are prevented, and reverted, not only during the exercise practice, but also during the activities of daily living.

The articles published in the 'Rehabilitation Schools for Scoliosis' Thematic series of the SCOLIOSIS journal will comply with some essential characteristics and format. The articles describing specific Rehabilitation Schools for Scoliosis should be organized as follows:

• **Introduction: **Includes general notes and goals of the study.

• **History: **A brief history of the Rehabilitation School.

• **Theoretical principles**: How the Rehabilitation program might work (theory). General description of the mechanical principles of correction for the various curve patterns (note classification used).

• **Exercises: **Detail specific exercises, with photos of how to perform them.

• **Practical issues**. This should be divided into the following parts:

○ **Protocols: **Description of the protocols generally used according to each clinical situation.

○ **Results & case reports: **Short review of published results. The results should be divided into groups related to short term (at least 6 months), midterm (end of growth) or long term (follow-up beyond growth). Also, the type of study performed (case series, controlled) and the population considered must be reported. Moreover, cases of different curve types, fully documented with photos, clinical data and radiographs, should be reported. However, patient consent must be obtained for this data, and provided upon submission.

○ **Discussion: **Comparison with other rehabilitation programs based on the author's hypothesis; strengths and limitations, advantages and disadvantages.

○ **Conclusions**: With final remarks.

○ **Abstract: **Organized with the following sections: background, rehabilitation program description and principles, results, conclusions. (indications for future research directions, technical notes concerning particular details of the rehabilitation program, devices used for the implementation of it, and so on.)

## Conclusion

SCOLIOSIS journal is focused on spinal deformities. Even though there is some evidence in favour of scoliosis specific exercises [1], the actual knowledge in the field does not yet allow us to classify the existing rehabilitation schools and methods. Consequently, to increase our collective knowledge in this field, we will publish in a systematic way, what is being done today by clinicians with the most expertise. Discussion must be open among these experts to allow progressive comparisons, and a deeper understanding, so their contributions will be accepted, and published, in the same thematic series of the journal, in parallel with the brace thematic series. We are confident that with this new effort, the journal will become an important source of information to the world of spinal deformity management, and will increase our understanding of how specific exercises affect the outcome of these problems. We do this for the benefit of the patients.

## Competing interests

The authors declare that they have no competing interests.

## Authors' contributions

MR and TBG contributed in the manuscript drafting. The authors read and approved the final manuscript.

## References

[B1] Negrini S Atanasio S Zaina F Romano M Rehabilitation of adolescent idiopathic scoliosis: results of exercises and bracing from a series of clinical studies. Europa Medicophysica-SIMFER 2007 Award Winner Eur J Phys Rehabil Med 2008 44 2 169 176 18418337

[B2] Weiss HR Negrini S Hawes CH Rigo M Kotwicki T Grivas TB Maruyama T members of the SOSORT Physical exercises in the treatment of idiopathic scoliosis at risk of brace treatment - SOSORT consensus paper 2005 Scoliosis 2006 1 6 (11 May 2006) 10.1186/1748-7161-1-6 16759360 PMC1481573

[B3] Dubousset J Importance of the three-dimensional concept in the treatment of scoliotic deformities In Dansereau J ed. International Symposium on 3D Scoliotic deformities joined with the VIIth International Symposium on Spinal Deformity and Surface Topography 1992 Edited in Germany, Gustav Fisher Verlag 302 311

[B4] Winter RB Lovell WW Moe JH Excessive thoracic lordosis and loss of pulmonary function in patients with idiopathic scoliosis J Bone Joint Surg Am 1975 56 7 972 977 1184646

[B5] Labelle H Dansereau J Bellefleur C Poitras B 3-D study of the immediate effect of the Boston brace on the scoliosis lumbar spine Ann Chir 1992 46 9 814 820 1299160

[B6] Willers U Nomelli H Aaro S Svensson O Hendlund R Long-term results of Boston brace treatment on vertebral rotation in idiopathic scoliosis Spine 1993 18 4 432 435 8470002

[B7] Labelle H Dansereau J Bellefleur C Poitras B Three-dimensional effect of the Boston brace on the thoracic spine and the rib cage Spine 1996 21 1 59 64 10.1097/00007632-199601010-00013 9122764

[B8] Wong MS Evans JH Biomechanical evaluation of the Milwaukee brace Prosthet Orthot Int 1998 22 1 54 67 9604276 10.3109/03093649809164457

[B9] Aubin CE Dansereau J de Guise JA Labelle H Rib cage-spine coupling patterns involved in brace treatment of adolescent idiopathic scoliosis Spine 1997 22 6 629 635 10.1097/00007632-199703150-00010 9089935

[B10] Stokes IA Three-dimensional terminology of spinal deformity: A report presented to the Scoliosis Research Society by the SRS Working Group on 3-D terminology of spinal deformity Spine 1994 19 236 248 10.1097/00007632-199401001-00020 8153835

[B11] Perdriolle R Vidal J Morphology of scoliosis: three-dimensional evolution Orthopedics 1987 10 6 909 915 3615285 10.3928/0147-7447-19870601-10

[B12] Perdriolle R Vidal J Thoracic idiopathic scoliosis curve evolution and progression Spine 1985 10 785 791 10.1097/00007632-198511000-00001 4089651

[B13] Asher M Cook LT D'Amico, Merolli, Santambrogio Adolescent idiopathic scoliosis transverse plane evolution. Threedimensional analysis of spinal deformities Health Technologies and Informatics 15 1995 IOS Press 253 258

[B14] Grivas TB Dangas S Samelis P Maziotou C Kandris Lateral spinal profile in school-screening referrals with and without late onset idiopathic scoliosis 10 degrees-20 degrees Stud Health Technol Inform 2002 91 25 31 15457689

[B15] Rigo M Quera-Salvá G Villagrasa M Sagittal configuration of the spine in girls with idiopathic scoliosis: progression rather than initiating factor Stud Health Technol Inform 2006 123 90 94 17108409

[B16] Burwell RG Aetiology of idiopathic scoliosis: current concepts Pediatric Rehabilitation 2003 6 3-4 137 170 14713582 10.1080/13638490310001642757

[B17] Burwell RG Dangerfield MH Freeman BJC Etiologic theories of idiopathic scoliosis. Somatic nervous system and the NOTOM escalator concept as one component in the pathogenesis of adolescent idiopathic scoliosis Stud Health Technol Inform 2008 140 208 217 18810026

[B18] Burwell RG Aujla RK Grevitt MP Dangerfield PH Moulton A Randfell TL Anderson SI Pathogenesis of adolescent idiopathic scoliosis in girls - a double neuro-osseous theory involving disharmony between two nervous systems, somatic and autonomic expressed in the spine and trunk: possible dependency on sympathetic nervous system and hormones with implications for medical therapy Scoliosis 2009 4 24 (Oct 31) 10.1186/1748-7161-4-24 19878575 PMC2781798

[B19] Stokes IA Burwell RG Dangerfield PH Biomechanical growth modulation and progressive adolescent scoliosis - a test of the 'vicious cycle' pathogenetic hypothesis: Summary of an electronic focus group debate of the IBSE Scoliosis 2006 1 16 10.1186/1748-7161-1-16 17049077 PMC1626075

[B20] Villemure I Aubin CE Dansereau J Labelle H Simulation of progressive deformities in adolescent idiopathic scoliosis using a biomechanical model integrating vertebral growth modulation J Biomech Eng 2002 124 6 784 790 10.1115/1.1516198 12596648

[B21] Castro FP Jr Adolescent idiopathic scoliosis, bracing and the Hueter-Volkmann principle Spine J 2003 3 3 180 105 10.1016/S1529-9430(02)00557-0 14589197

[B22] Haderspeck K Shultz A Progression of idiopathic scoliosis: an analysis of muscle action and body weiht influences Spine 1981 6 5 447 455 10.1097/00007632-198109000-00005 7302678

[B23] Schultz AB Dansereau J Threedimensional model analyses of scoliosis biomechanics International symposium 3D scoliotic deformities 1992 Éditions de l'École Polytechnique de Montréal. Gustav Fisher Verlag 62 70

[B24] Shimada Y A study of trunk miscle in idiopathic scoliosis Nippon Seikeigeka Gakkai Zasshi 1989 63 1 33 34 2723496

[B25] Cheung J Veldhuizen AG Halbertsma JP Maurits NM Sluiter WJ Cool JC Van Horn JR The relation between electromyography and growth velocity of spine in the evaluation of curve progression in idiopathic scoliosis Spine 2004 29 9 1011 1016 10.1097/00007632-200405010-00012 15105674

[B26] Cheung J Veldhuizen AG Halberts JP Sluiter WJ Van Horn JR Geometric and electromyographic assessments in the evaluation of curve progression in idiopathic scoliosis Spine 2006 31 3 322 329 10.1097/01.brs.0000197155.68983.d8 16449906

[B27] Dickson RA Lawton JO Archer IA Butt WP The pathogenesis of idiopathic scoliosis. Biplanar spinal asymmetry J Bone Joint Surg Br 1984 66 1 8 15 6693483 10.1302/0301-620X.66B1.6693483

[B28] Burwell RG Cole AA Cook TA Grivas TB Kiel AW Moulton A Thirlwall AS Upahhyaya SS Webb JK Wemyss-Holden SA The pathogenesis of idiopathic scoliosis: The Nottingham concept Acta Orthopaedica Belgica 1992 58 33 58 1456018

[B29] Guo X Chau WW Chan YL Cheng JC Burwell RG Dangerfield PH Relative anterior spinal overgrowth in adolescent idiopathic scoliosis- results of disproportionate endochondral-membranous bone growth?. Summary of an electronic focus group debate of the IBSE Eur Spine J 2005 14 9 862 73 10.1007/s00586-005-1002-7 16133084

[B30] Grivas TB Vasiliades E Malakasis M Mouzakis V Segos D Intervertebral disc biomechanics in the pathogenesis of idiopathic scoliosis6th Biennial Meeting of the International Research Society of Spinal Deformities (IRSSD) 2006 The Cultural and Congress Centre of the University of Ghent'Het Pand', Ghent, Belgium

[B31] Grivas TB Vasiliadis E Malakasis M Mouzakis V Segos D Intervertebral disc biomechanics in the pathogenesis of idiopathic scoliosis Stud Health Technol Inform 2006 123 80 83 17108407

[B32] Will RE Stokes IA Qin X Walker MR Sanders JO Cobb angle progression in adolescent scoliosis begins at the intervertebral disc Spine 2009 34 25 2782 2786 10.1097/BRS.0b013e3181c11853 19940737 PMC2784923

[B33] Melrose J Gurr KR Cole TC Darvodelsky A Ghosh P Taylor TK The influence of scoliosis and ageing on proteoglycan heterogeneity in the human intervertebral disc J Orthop Res 1991 9 1 68 77 10.1002/jor.1100090110 1984051

[B34] Taylor TKF Melrose J Burwell G, Dangerfield PH, Lowe TG, Margulies YJ The role of the intervertebral disc in adolescent idiopathic scoliosis Etiology of adolescent idiopathic scoliosis: Current trends and relevance to new treatment approaches 2000 Philadelphia:Hanley and Belfus Inc

[B35] Bushell GR Ghosh P Taylor TK Sutherland JM The collagen of the intervertebral disc in adolescent idiopathic scoliosis J Bone Joint Surg Br 1979 61B 4 501 508 10.1302/0301-620X.61B4.500764 500764

[B36] Dangerfield PH Roberts N D'Amico M, Santambrogio GC, Merolli A Investigation of the diurnal variation in the water content of the intervertebral disc using MRI and its implication for scoliosis Three-dimensional analysis of spinal deformities 1995 Amsterdam: IOS Press 447 451

[B37] Grivas TB Vasiliadis ES Dodopoulos G Bardakos N Gatos C The role of the intervertebral disc in correction of scoliotic curves. A theorical model of idiopathic scoliosis pathogenesis Stud Health Technol Inform 2008 140 33 36 18809995

